# Mothers’ psychopathology and their adult offspring’s cortisol level in a Rwandan sample

**DOI:** 10.4102/sajpsychiatry.v31i0.2410

**Published:** 2025-05-15

**Authors:** Marie C. Ingabire, Serge Caparos, Eugène Rutembesa, Isabelle Blanchette

**Affiliations:** 1 Département de Psychologie, Université du Québec à Trois-Rivières, Trois-Rivières, Québec, Canada; 2 Département de Psychologie, Université Paris 8, Paris, France; 3 Institut Universitaire de France, Paris, France; 4 Department of Psychiatry and Behavioral Sciences, University of Rwanda, Kigali, Rwanda; 5 École de Psychologie, Faculté des Sciences Sociales, Université Laval, Québec, Canada

**Keywords:** PTSD, depression, psychopathology, cortisol, intergenerational transmission of trauma

## Abstract

**Background:**

Most studies on the influence of mothers’ trauma-related psychopathology on their offspring’s hypothalamic-pituitary-adrenal (HPA) axis functioning have been conducted in Western contexts. Furthermore, those studies have focused on the association between mothers’ post-traumatic stress disorder (PTSD) and their offspring’s HPA axis functioning. More research is needed among African populations exposed to mass violence to mitigate the intergenerational transmission of trauma.

**Aim:**

To investigate the link between mothers’ PTSD and depression and their offspring’s basal cortisol level.

**Setting:**

This cross-sectional study was conducted in two provinces of Rwanda (Kigali City and the Southern Province) among families of survivors of the 1994 genocide perpetrated against the Tutsi.

**Methods:**

A total of 45 dyads of mothers and their adult offspring were recruited. They answered questionnaires that measured sociodemographic characteristics, trauma exposure, PTSD and depression symptoms. Participants also provided saliva samples for cortisol extraction.

**Results:**

Mothers’ depression was negatively associated with their offspring’s overall basal cortisol level. There was no link between mothers’ PTSD and their offspring’s overall basal cortisol level. The relationship between the offspring’s overall basal cortisol level and their own psychopathology was not significant.

**Conclusion:**

These preliminary findings showed an HPA axis disruption among offspring of mass violence-exposed and depressed mothers.

**Contribution:**

This study contributes to the literature by showing that depression is a relevant correlate of neuroendocrine functioning and should be investigated more consistently in research on the intergenerational consequences of trauma exposure.

## Introduction

Africa is plagued by instances of mass violence, such as armed conflicts and ethnic cleansing, that expose the civil populations to traumatic events. Exposure to traumatic events can trigger the onset of post-traumatic stress disorder (PTSD) and depression.^1,2^ Furthermore, PTSD in trauma-exposed parents is a risk factor for psychopathological disorders among their offspring.^3^ The dysfunction of the hypothalamic-pituitary-adrenal (HPA) axis, as reflected in altered cortisol (a glucocorticoid stress-regulating hormone) levels, has been proposed as a transmission mechanism that underlies the intergenerational transmission of trauma.^4,5^ Most of the studies on this topic were conducted among the children of Holocaust survivors and focused primarily on maternal PTSD.^6,7^ In this study, we sought to extend this line of research to an African sample by investigating the relationship between mothers’ PTSD and depression, and their adult offspring’s cortisol levels in Rwanda. A better understanding of the transmission mechanisms of trauma may contribute to its prevention.

### The genocide against the Tutsi and related mental health problems

The 1994 genocide perpetrated against the Tutsi in Rwanda cost the lives of around a million people and exposed the population to traumatic events such as the death of loved ones and sexual violence.^8^ This exposure has been associated with mental health problems. A recent meta-analysis found a PTSD rate of 37% among genocide survivors.^9^ Depression was similarly prevalent, affecting 35% - 48%^10,11^ of genocide survivors. In addition, a national study conducted in Rwanda 14 years after the genocide found that 68% of individuals with PTSD also had comorbid depression.^2^

### Intergenerational transmission of trauma and underlying mechanisms

Parents’ PTSD, particularly mothers’ PTSD, has been associated with an elevated risk of PTSD and other psychopathological disorders among children of Holocaust survivors^12,13^ and children of war veterans.^14^ Similarly, the offspring of women exposed to the genocide in Rwanda during pregnancy had more severe PTSD and depression symptoms than those of non-exposed women.^4^ However, some studies conducted in Rwanda and internationally found no association between mothers’ PTSD and their offspring’s psychopathological symptoms.^15,16^

Psychological^17^ and epigenetic mechanisms^18^ may underlie the intergenerational transmission of trauma. With regard to epigenetic mechanisms, mothers with PTSD display disruptions in the functioning of the HPA axis, as evidenced by lower basal cortisol levels,^6,19,20^ and these alterations may be transmitted to their offspring through the expression of stress-regulating genes or HPA axis programming in utero.^4,21^ Typically, the release of optimal cortisol levels is responsible for an adaptative psychophysiological response to stressful and traumatic events.^22^ Therefore, non-optimal cortisol levels can contribute to the development of psychopathology.^22,23^

Several studies have shown lower basal cortisol levels among offspring of Holocaust survivors whose parents had PTSD in comparison to control groups of Jews.^6,24,25^ Similar findings were reported among infants whose mothers developed PTSD following exposure to the 9/11 terrorist attack in the United States of America (US),^26^ and offspring of Rwandan mothers exposed to the genocide during pregnancy.^4^ In turn, the offspring’s lower basal cortisol levels were associated with a higher PTSD symptom severity.^4,24,25^ However, in some studies, no link was found between parents’ PTSD and their offspring’s basal cortisol levels.^27^

### Intergenerational transmission of trauma and depression

Most studies have focused on the intergenerational influence of maternal PTSD.^20^ However, extensive evidence shows a high comorbidity of PTSD and depression among trauma-exposed individuals.^2,28^ In addition, depression is associated with a reverse cortisol profile, whereby individuals who suffer from depression and their children exhibit higher basal cortisol levels than non-depressed individuals.^29,30^ Accounting for mothers’ comorbid depression may contribute to explaining the divergent results observed in studies on the relationship between mothers’ PTSD and children’s cortisol levels.^24,31^

A few studies have investigated the association between maternal depression and their offspring’s cortisol levels. Among samples of Holocaust survivors and American women exposed to the 9/11 terrorist attack, maternal depression was not associated with children’s basal cortisol levels.^24,26^ In a study of war veterans, long-term basal cortisol levels were higher among offspring whose fathers had both PTSD and depression than those whose fathers only had PTSD.^27^ In studies that did not measure trauma exposure and PTSD, mothers’ depression was related to higher basal cortisol levels among their offspring compared to control groups.^30,32^ So far, studies on this question are relatively few and have yielded mixed results.

Additional research is needed to understand better the link between mothers’ PTSD and depression and offspring’s cortisol level among trauma-exposed populations. This field of investigation is quasi-inexistent in Africa despite a high occurrence of mass violence on that continent.^33^ The context in which people live, such as living conditions (e.g. security levels, poverty), lifestyle and nutrition, can influence cortisol levels.^34,35,36^ Enhanced comprehension of neuroendocrine risk factors for stress-related disorders may inform future avenues for preventing and treating those disorders.

This study aimed to contribute to the literature by examining the relationship between mothers’ PTSD and depression and their offspring’s basal cortisol level in a sample of genocide survivors, where children were conceived and born after the genocide. Based on prior research, we hypothesised that (1) mothers’ PTSD would be negatively linked to their offspring’s basal cortisol level; (2) mothers’ depression would be positively linked to their offspring’s basal cortisol level and (3) offspring’s basal cortisol level would be negatively linked to their own PTSD symptoms and positively linked to their own depression symptoms.

## Research methods and design

### Study design

This cross-sectional study was conducted among families of survivors of the 1994 genocide against the Tutsi. The study was part of a larger project examining the intergenerational transmission of trauma in Rwanda. We previously reported, among the full sample of 181 dyads, the link between mothers’ trauma exposure and PTSD, and their offspring’ psychopathology, cognitive functioning and attitudes towards reconciliation.^37,38^

### Setting

Participants in this study were recruited from several communities in two provinces of Rwanda, namely Kigali City (69%) and the Southern province (31%).

### Study population and sampling

The study population was genocide survivors. The sample of 45 mother-child dyads was randomly selected among the participants of a larger sample of 181 dyads (recruited conveniently). If a dyad declined the invitation to provide saliva samples (10 out of 181 dyads declined), the recruitment continued until the targeted sample size was reached. The sample size was determined based on previous similar studies^4,26^ and financial resource constraints. The inclusion criteria were as follows: (1) mothers had to have been targeted by the genocide against the Tutsi; (2) they had to be the biological mothers of the child participating in the study; and (3) offspring had to be born after the genocide (1995–2000). Exclusion criteria were as follows: (1) having a severe psychiatric disorder (e.g. psychotic or bipolar disorder), and/or neurological disorder; (2) having a chronic medical condition or an illness requiring medication at the time of the study (e.g. human immunodeficiency virus [HIV]/acquired immunodeficiency syndrome [AIDS]; bacterial infection); (3) smoking cigarettes or any other psychoactive substances (e.g. marijuana); and (4) excessive alcohol consumption (self-reported or diagnosed by a health professional).

### Data collection

Data were collected in July and August 2018 and 2019 by the first author and an experienced research assistant. A mother and her child came together to the research venue but completed the questionnaires in two separate rooms to ensure confidentiality. All questionnaires were translated into Kinyarwanda, the local language and presented on a computer using E-Prime software. Participants took home the tubes for collecting saliva and submitted them to the first author once saliva was collected.

### Instruments

#### Sociodemographic questionnaire

A socio-demographic questionnaire previously used in Rwanda^39^ was used to gather information about age, sex, education, marital status, socioeconomic status, family size and living arrangements.

#### Harvard Trauma Questionnaire

The Harvard Trauma Questionnaire, Part I (HTQ)^40^ was used to assess trauma exposure. The questionnaire comprised 28 potentially traumatic events (e.g. beating to the body, witnessing murder). Participants indicated whether they had experienced each event. Mothers answered the questionnaire about the events of the genocide and offspring (who did not experience the genocide) in relation to their lifetime. We computed a total percentage score for each participant and used it as an index of trauma exposure severity. The HTQ has been widely used in various post-conflict countries, including Rwanda, and has sound psychometric qualities.^41^

#### Post-traumatic Stress Disorder Checklist-5

The PTSD Checklist, version 5 (PCL-5),^42^ a 20-item scale based on the Diagnostic and Statistical Manual of Mental Disorders, Fifth Edition (DSM-5) criteria for PTSD,^43^ was used to measure PTSD symptoms. Participants indicated whether they had experienced each symptom in the previous month (e.g. feeling very upset when something reminds the person of the stressful experience; trouble falling or staying asleep) on a 5-point scale ranging from 0 (*not at all*) to 4 (*extremely*). We calculated a total score for each participant (possible range: 0–80), with higher scores indicating a higher PTSD symptom severity. A cut-off score of 33 was used to determine probable PTSD.^44^ The PCL-5 has been widely used in post-conflict settings and has shown good psychometric qualities.^44,45^ The Cronbach alpha was 0.93 in this entire sample, 0.90 among mothers and 0.93 among the offspring.

#### Hopkins Symptom Checklist

The Hopkins Symptom Checklist (HSC)^46^ was used to measure symptoms of depression in the previous week. The depression subscale comprised 15 items (e.g. feeling no interest in things, thoughts of ending one’s life) rated on a scale ranging from 1 (*not at all*) to 4 *(extremely*). A total percentage score was calculated for each participant, with higher scores indicating higher symptom severity. A mean percentage cut-off score of 55 was used to determine probable depression.^47^ The HSC has been previously used in Rwanda and had good psychometric properties.^48^ Herein, the scale showed a good internal consistency with a Cronbach alpha of 0.93 in the entire sample, 0.93 among mothers and 0.91 among the offspring.

#### Saliva collection and cortisol extraction

Saliva was collected, and cortisol was extracted following procedures used in previous studies.^49,50^ Each participant collected six saliva samples on 2 consecutive days (3 times a day), immediately after waking up, 30 m after waking up and before bedtime. In the morning, participants collected saliva before brushing their teeth and consuming any meal. In the evening, they collected the saliva at least 1 h and a half after the last meal. Participants were instructed to collect saliva on days they did not expect to be stressful to obtain accurate basal cortisol levels. Each participant was given a sheet for logging saliva collection times. Saliva samples were collected in 5 mL Sarstedt salivette tubes. Because participants had no access to freezers in their homes, they were instructed to keep the samples in a clean, dry and cool place. Samples were then transported to the BioAssay Laboratory of the Centre for Studies on Human Stress in Montreal, Canada, where they were frozen at −20°C. For cortisol extraction, samples were brought to room temperature, centrifuged at 3000 rpm for 15 m and analysed using a high-sensitivity enzyme immunoassay procedure. Two duplicates were run in every essay, and the mean intraassay coefficient of variation (CV) was 15.2%. To determine the overall basal cortisol level, we computed the mean of the 6 six samples collected over 2 days; the overall cortisol level was then used in subsequent analyses.

### Data analysis

Data were transposed from Excel to IBM Statistical Package for the Social Sciences (SPSS) version 29 (IBM Corp., Released 2023, Armonk, NY) for analysis. Data cleaning showed that cortisol data were positively skewed; a log transformation was used to correct the data distribution. Given the relatively small size of our sample, deviation from a normal distribution (trauma exposure, PTSD and depression) and the presence of outliers, we also used a bias-corrected and accelerated bootstrap with 5000 samples and 95% confidence intervals (CIs).^51^ The data (total scores; *n* = 90) were missing as follows: trauma exposure: 6.6%, PTSD: 10%, depression: 2.2% and cortisol levels (mean score): 4.4%. The multiple imputation method was used to account for missing data. Means (with standard deviations) and frequency analyses were used to describe the data. We ran two independent *t-* tests to test the influence of mothers’ PTSD (PTSD compared to without PTSD) and depression (depression compared to without depression) on their offspring’s cortisol levels. We also examined the relationship between offspring’s cortisol levels and mothers’ level of PTSD and depression symptoms as continuous variables by performing correlational analyses. All analyses involving cortisol levels were performed on log-transformed data; however, we report raw values for ease of interpretation.

### Ethical considerations

Each participant was given an information letter detailing the study and its objectives. Participants were offered verbal explanations when needed. Participants gave written consent for participating in the study in general and specific consent about providing saliva samples. Each participant received 14 000 FRW (~20$ CAD) for their time. Ethical clearance to conduct this study was obtained from the Republic of Rwanda National Ethics Committee (No. 165/RNEC/2018).

## Results

The description of the participants’ sociodemographic data is presented in Table 1. Mothers’ mean age was 45.13 years (standard deviation [s.d.] = 6.21; range: 35–62 years), and offspring’s mean age was 20.27 years (s.d. = 1.75; range: 18–24 years). All offspring were single, and 91% (*n* = 41) lived in the same household as their mothers. Most households (*n* = 32; 71%) reported a modest socioeconomic status.

**TABLE 1:** Sociodemographic characteristics of the participants.

Characteristic	Mothers		Offspring	

	*n*	%	*n*	%
**Sex**				
Female	45	100.0	25	55.6
Male	-	-	20	44.4
**Education**				
No education	2	4.4	-	-
Primary education	18	40.0	5	11.1
Incomplete secondary education or vocational training	14	31.1	15	33.3
Complete secondary education	5	11.1	20	44.4
University education	6	13.3	5	11.1
**Marital status**				
Married or in a partnership	23	51.1	-	-
Widowed	10	22.2	-	-
Separated or divorced	9	20.0	-	-
Single	3	6.7	45	100.0
**Economic status**				
Very modest	9	20.0	-	-
Modest	13	28.9	-	-
A little bit modest	10	22.2	-	-
Neither modest nor well off	9	20.0	-	-
A bit well off	4	8.9	-	-
**Mother-child living together**				
Yes	41	91.1	-	-

Participants’ mean scores of trauma exposure, PTSD and depression symptoms and untransformed cortisol levels are summarised in Table 2. Mothers had significantly higher levels of trauma exposure, *t*(88) = 15.41 (bias-corrected and accelerated [BCa] 95% CI: 13.42 to 17.35), *p* < 0.001; PTSD symptoms, *t*(88) = 5.09 (BCa 95% CI: 3.12 to 7.04), *p* < 0.001 and depression symptoms, *t*(88) = 3.82 (BCa 95% CI: 1.89 to 5.82), *p* < 0.001 compared to their offspring (see Table 2 for descriptive statistics). In all, 21 mothers (47%) and 8 offspring (18%) met the criteria for probable PTSD; 15 mothers (33%) and 5 offspring (11%) scored above the cut-off score for probable depression.

**TABLE 2:** Cortisol levels and psychopathological symptom severity (*N* = 90).

Variables	Mothers (*n* = 45)			Offspring (*n* = 45)		
	*M*	BCa 95% CI	s.d.	*M*	BCa 95% CI	s.d.
Trauma exposure	66.35	61.37–71.11	16.25	14.71	10.23–19.74	16.23
PTSD symptoms	32.76	29.97–36.48	13.39	17.86	13.89–22.22	14.65
Depression symptoms	46.14	39.03–53.16	23.71	27.69	21.66–34.01	20.94
Cortisol awakening†	1.24	0.91–1.16	1.27	1.73	1.15–2.39	2.17
Cortisol 30 min after awakening?	1.28	0.88–1.70	1.57	1.92	1.25–2.75	2.60
Cortisol bedtime?	0.69	0.54–0.88	0.63	0.79	0.57–1.10	0.97
Cortisol overall?	1.07	0.82–1.35	0.99	1.45	1.03–1.96	1.59

Note: Cortisol levels are measured in nmol/L.

† , average of cortisol levels over 2 days.

M, mean; s.d., standard deviation; BCa, bias-corrected and accelerated; CI, confidence interval; min, minutes; PTSD, post-traumatic stress disorder.

**FIGURE 1:**
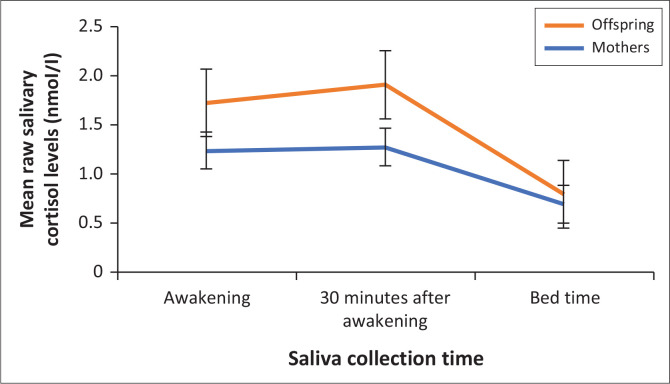
Circadian pattern of mothers’ and offspring’s cortisol levels.

Cortisol levels of both mothers and offspring followed the typical circadian rhythm, with higher levels in the morning and lower levels at bedtime (see Figure 1). Among offspring, the overall cortisol level of men, *M* = 1.39, s.d. = 1.73, did not differ significantly from that of women, *M* = 1.50, s.d. = 1.51; *t*(43) = −1.04 (BCa 95% CI: −2.93 to 0.92), *p* = 0.29. Correlations between cortisol levels of mothers and their offspring are presented in Table 3. Overall, cortisol levels upon awakening and 30 m later correlated moderately or highly. There was a small or no correlation between bedtime cortisol levels and cortisol levels at awakening and 30 m after awakening. There was no correlation between age and overall cortisol level among offspring, *r* = −0.20, (BCa 95% CI: −0.44 to 0.05), *p* = 0.18 or among mothers, *r* = −0.13 (BCa 95% CI: −0.34 to 0.12), *p* = 0.39. Furthermore, mothers’ overall cortisol level did not differ significantly from their offspring’s overall cortisol level, *t*(88) = −0.90 (BCa 95% CI: −0.25 to 1.02), *p* = 0.37 (see Table 2 for descriptive statistics).

**TABLE 3:** Correlations between cortisol levels at different time points.

Cortisol levels	T1D1	T2D1	T3D1	Mean D1	T1 D2	T2 D2	T3 D2	Mean D2
T1D1	-	0.76**	0.54**	0.92**	0.75**	0.64**	0.19	0.81**
T2D1	0.51**	-	0.44*	0.87**	0.60**	0.60**	0.07	0.67*
T3D1	0.19	0.32*	-	0.68**	0.56**	0.54**	0.42*	0.67**
Mean D1	0.76**	0.82**	0.37*	-	0.78**	0.67**	0.22	0.82**
T1D2	0.41*	0.57**	0.22	0.53**	-	0.59**	0.04	0.83**
T2D2	0.52**	0.62**	0.09	0.54**	0.57**	-	0.15	0.87**
T3D2	0.35*	0.55**	0.28*	0.54**	0.21	0.41*	-	0.25
Mean D2	0.54**	0.75**	0.30*	0.68**	0.83**	0.54**	0.54**	-

Note: Offspring data (*N* = 45) are presented above the diagonal line; mothers’ data (*N* = 45) are presented below the diagonal line.

T1, cortisol level at awakening; T2, cortisol level 30 min after awakening; T3, cortisol level at bedtime; D1, day 1; D2, day 2; Mean D1, average of cortisol measures on day 1; Mean D2, average of cortisol measures on day 2.

* , *p* < 0.05;

** , *p* < 0.001.

### Associations between psychopathology and cortisol levels in each generation

Among offspring, trauma exposure correlated with both PTSD symptoms, *r* = 0.32 (BCa 95% CI: 0.10 to 0.62), *p* = 0.03, and depression symptoms, *r* = 0.38 (BCa 95% CI: 0.12 to 0.63), *p* = 0.01. Post-traumatic stress disorder symptoms highly correlated with depression symptoms, *r* = 0.71, *p* < 0.001 (BCa 95% CI: 0.50 to 0.87). The overall cortisol level did not correlate with trauma exposure, *r* = 0.05 (BCa 95% CI: −0.29 to 0.28), *p* = 0.72; PTSD symptoms *r* = −0.06 (BCa 95% CI: −0.28 to 0.17), *p* = 0.67 or depression symptoms, *r* = −0.17 (BCa 95 % CI −0.38 to 0.07), *p* = 0.26. Among mothers, trauma exposure did not correlate significantly with PTSD symptoms, *r* = 0.22 (BCa 95% CI: −0.08 to 0.47), *p* = 0.14 or depression symptoms, *r* = 0.15 (BCa 95% CI: −0.16 to 0.44), *p* = 0.31. There was a high correlation between PTSD symptoms and depression symptoms, *r* = 0.81, (BCa 95% CI: 0.72 to 0.88), *p* < 0.001. The overall cortisol level did not correlate with trauma exposure, *r* = 0.08 (BCa 95% CI: −0.17 to 0.31), *p* = 0.59; PTSD symptoms, *r* = 0.07 (BCa 95% CI: −0.36 to 0.22), *p* = 0.66 or depression symptoms, *r* = −0.21 (BCa 95% CI: −0.49 to 0.08), *p* = 0.17.

### Associations between mothers’ post-traumatic stress disorder and depression and their offspring’s overall cortisol level

Offspring of mothers with depression had a lower overall cortisol level (*n* = 15; *M* = 0.88, s.d. = 0.87) than the offspring of mothers without depression (*n* = 30; *M* = 1.74, s.d. = 1.79), *t*(43) = 2.33 (BCa 95% CI: 0.41 to 4.30), *p* = 0.03, Cohen’s *d* = 0.80. The results showed no significant differences in the overall cortisol level between the offspring of mothers with PTSD (n = 21; M = 1.28, s.d. = 1.22) and those without PTSD (*n* = 24; *M* = 1.60, s.d. = 1.86), *t*(43) = 0.45 (BCa 95% CI: −1.45 to 2.46), *p* = 0.65, Cohen’s *d* = 0.13. In correlational analyses, mothers’ PTSD symptoms did not correlate with their offspring’s overall cortisol level, *r* = −0.15 (BCa 95% CI: −0.37 to 0.10), *p* = 0.33, but mothers’ depression symptoms negatively correlated with their offspring’s overall cortisol level, *r* = −0.36 (BCa 95% CI: −0.57 to −0.07), *p* = 0.02. Furthermore, partial correlations showed that the relationship between mothers’ depression symptoms and their offspring’s overall cortisol level remained significant after controlling for mothers’ PTSD, *r* = −0.41 (BCa 95% CI: −0.61 to −0.16), *p* = 0.005 and mothers’ trauma exposure, *r* = −0.33 (BCa 95% CI: −0.56 to −0.07), *p* = 0.03.

## Discussion

The primary objective of this study was to examine the intergenerational link between mothers’ PTSD and depression and their offspring’s basal cortisol level and whether the offspring’s basal cortisol level was linked to their own psychopathology. The findings revealed a negative link between mothers’ depression and their offspring’s basal cortisol level but not between mothers’ PTSD and their offspring’s cortisol level. No association was found between the offspring’s basal cortisol level and their own PTSD or depression symptoms.

The findings showed that mothers’ depression was associated with a lower overall cortisol level among their offspring. To our knowledge, this is the first study to find a negative relationship between mothers’ depression and offspring’s cortisol level in an African sample. This result contrasts with previous studies that reported higher basal cortisol levels in children of mothers suffering from depression.^30,32^ However, those studies are different in that they did not measure trauma exposure or the presence of PTSD. Among trauma-exposed parents, some studies reported no link between mothers’ depression and their offspring’s basal cortisol levels in samples of Holocaust survivors and American mothers exposed to the 9/11 terrorist attack in the US.^24,26^ Research on this topic in the context of mass violence is still sparse. The results of this study are preliminary and suggest that maternal depression should be more consistently investigated in studies on the intergenerational consequences of trauma.

Contrary to previous studies,^4,6^ we found no link between mothers’ PTSD and their offspring’s overall cortisol level. A previous Rwandan study showed lower basal cortisol levels among offspring whose mothers were exposed to genocide during pregnancy compared to offspring of non-exposed mothers.^4^ In our study, offspring were born after the genocide. It is plausible that maternal trauma exposure is more detrimental to their children when it occurs during pregnancy.^26^ Another difference is that the previous study^4^ compared the offspring of genocide-exposed to those of non-exposed mothers. In contrast, this study compared the cortisol levels of offspring whose mothers were exposed to the genocide and only differed in terms of PTSD severity. These differences may explain the divergent results of the two studies.

The results showed no link between offspring’s overall basal cortisol level and their PTSD or depression symptoms. This result is consistent with the current literature’s mixed findings on HPA axis dysfunction and trauma-related psychopathology.^52^ While some studies have reported lower basal cortisol in people with PTSD^53^ and higher basal cortisol levels in individuals with depression,^54^ other studies did not find a relationship between cortisol level and PTSD^52,55^ or depression.^56,57^ These inconsistent results may be attributed to methodological differences.^53,58^ For example, we assessed salivary cortisol, whereas a previous Rwandan study^4^ measured plasma cortisol. Standardising research protocols may help determine the sources of variation across studies.^58^

## Limitations

This study is not without limitations. Although comparable to those of earlier studies,^4,6^ our sample size was small and not representative of genocide survivors. Future studies should include larger samples that allow for a more adequate accounting of the interindividual variability of cortisol levels.^55^ Future studies, including larger samples, should also assess whether there is an interaction between mothers’ PTSD and depression on their offspring’s cortisol levels. Another limitation is that we did not control for potential confounding variables, such as body mass index, physical activity, sleep quality, menstrual cycles and current stress levels, that may influence cortisol levels.^54,59^ Furthermore, because of the lack of freezers in participants’ homes, saliva samples were kept at ambient temperatures before being frozen. This might be the reason why cortisol concentrations were lower than those typically reported.^26^ Nevertheless, the observation of a typical circadian rhythm of cortisol provides confidence that the cortisol data can be reliably interpreted.

## Conclusion

In conclusion, this study contributes to the literature by examining the mechanisms underlying the intergenerational transmission of trauma in an African sample. The findings showed a relationship between mothers’ depression, but not PTSD, and their adult offspring’s basal cortisol level. These results suggest that, in addition to PTSD, more empirical attention should be devoted to maternal depression in the study of intergenerational consequences of trauma. Considering the heterogeneity of the findings on the relationship between cortisol levels and trauma-related psychopathology, there is a need for standardising research protocols. More research on neuroendocrine correlates of trauma-related psychopathology is needed among the African populations, given the high occurrence of mass violence in Africa. This knowledge may aid in preventing the transmission of trauma from one generation to the next and in elaborating therapeutic interventions.
